# Prognostic significance of lymphangiogenesis in pharyngolaryngeal carcinoma patients

**DOI:** 10.1186/1471-2407-10-416

**Published:** 2010-08-10

**Authors:** Darío Garcia-Carracedo, Juan Pablo Rodrigo, Aurora Astudillo, Carlos Suarez Nieto, Maria Victoria Gonzalez

**Affiliations:** 1 Instituto Universitario de Oncología del Principado de Asturias (IUOPA), Universidad de Oviedo, Asturias, Spain; 2 Servicio de Otorrinolaringología, Hospital Universitario Central de Asturias (HUCA), Oviedo (Asturias), Spain; 3 Servicio Anatomía Patológica, Hospital Universitario Central de Asturias (HUCA), Oviedo (Asturias), Spain

## Abstract

**Background:**

Lymphatic vessel spread is considered a major route for head and neck squamous cell carcinoma metastasis. Formation of new lymphatic vessels could facilitate the process, raising the malignant potential of these tumours. Recent identification of lymphatic markers allows the study of the lymphangiogenesis phenomenon. We searched for molecular events involved in the lymphangiogenic process that could have prognostic value in laryngeal/pharyngeal carcinoma patients.

**Methods:**

104 paraffin-embedded pharyngeal/laryngeal tumour samples were studied. Immunohistochemical analysis of podoplanin and double immunofluorescence analysis of Ki-67 and D2-40 were performed. Lymph vessel density (inside the tumour mass, at its periphery or considered as a whole) and the presence of tumour emboli inside lymphatics were recorded. The proliferative state of endothelial lymphatic cells was evaluated.

**Results:**

Lymphatic vessels were detected inside the tumour mass (75%) and in the surrounding tissue (80%); some of them in a proliferative state. Tumour emboli were detected in a high proportion of the cases (45%). Lymphatic vessel density was higher in the pharyngeal cases (p = 0.0029), in greater size (p = 0.039), more advanced stage primary tumours (p = 0.006) and in carcinomas of patients with affected nodes (p = 0.019). The presence of tumour emboli and a high global vessel density were indicators of poor prognosis (recorded as death from tumour) in the laryngeal group (p = 0.015 and p = 0.027, respectively), but notably not in the pharyngeal one. Interestingly, high global vessel density showed a negative prognostic value among pathologically staged N0 laryngeal carcinomas (p = 0.03).

**Conclusions:**

The lymphangiogenic process correlated with aggressive tumour features (pN category, tumour size, tumour stage), but might play different roles in tumours arising from different anatomic sites.

Our results suggest that detection of tumour emboli and assessment of global vessel density using the D2-40 antibody, may be useful in the clinical practice, as predictors of reduced survival among pN0 laryngeal carcinoma patients.

## Background

Head and neck squamous cell carcinoma (HNSCC) remains a significant cause of morbidity and mortality, afflicting 500.000 new cases worldwide each year[1]. The single most adverse independent prognostic factor for patients with HNSCC is the involvement of regional lymph nodes, but its accuracy could be improved.

Invasion of cells into the surrounding tissue and the destruction of normal tissue architecture are two hallmarks of malignant tumours. Lymphatic vessels serve as the primary conduit for malignant tumour cell to regional lymph nodes[2]. In recent years, increasing evidence support that lymphangiogenesis is involved in the process of lymphatic spread in HNSCC. However, whether cancer cells can metastasize by expansion and invasion of preexisting peritumoural lymphatics, or by the formation and invasion of new lymphatics within tumours remains an unsolved question due to the difficulty in distinguishing lymphatics from blood vessels[3-8]. Recently, several lymphatic endothelium markers were identified and the most reliable one among them is podoplanin, which is recognised by the monoclonal D2-40 antibody with a high specificity and sensitivity [9-11].

Using the D2-40 antibody in this study, the presence and density of lymphatic vessels were determined and quantified in laryngeal/pharyngeal carcinomas, and their potential prognostic values were assessed.

## Methods

### Patients

Paraffin-embedded tissues from 104 patients with pharyngeal or laryngeal squamous cell carcinoma (Table 1) who underwent resection of their tumours at the Hospital Universitario Central de Asturias (HUCA) (2000 - 2006) were obtained from the Pathology Department. Having a balanced number of cases for every clinicopathologic category was one of the patient inclusion criteria. Prior to the start the study was evaluated for approval according to the institutional review board's guidelines on ethical procedures. None of the patients had received radio/chemotherapy prior to resection or were thought to have distant metastasis at the time of diagnosis. Mean follow-up periods are shown in Table 1. Endpoints examined were nodal involvement, disease specific and overall survival.

**Table 1:** Clinicopathologic features of the laryngeal/pharyngeal squamous cell carcinoma patients and their primary tumours (N= 104).

Clinicopathologic feature	N° cases (%)
**Age**	Mean 60 (33-86)

**Sex**	
Male	98 (94.2)
Female	6 (5.8)

**Tumour site**	
Hypopharynx	18 (17.5)
Oropharynx	31 (30.1)
Glottis	30 (29.1)
Supraglottis	24 (23.3)

**T category**	
1	19 (18.3)
2	20 (19.2)
3	30 (28.9)
4	35 (33.6)

**N category**	
0	47 (45.2)
1	8 (7.7)
2	41 (39.4)
3	8 (7.7)

**Degree of differentiation**	
Well	48 (49)
Moderate	32 (32.6)
Poor	18 (18.4)

**Tumour stage (TNM^a^)**	
I	18 (18.18)
II	10 (10.10)
III	14 (14.14)
IV	57 (57.58)

**Status**	
Alive without tumour	49 (47)
Alive with tumour	3 (3)
Death of tumour	34 (33)
Death other causes	18 (17)

**Recurrence**	
No	66 (66)
Yes	34 (34)

**Toxic habits**	
Cigarette smoking	103 (99)
Alcohol consumption	91 (88)

**Mean follow up (months) (range)**	
Whole population	23 (10 - 67)
Larynx	26 (9 - 67)
Pharynx	20 (10 - 66)

^a^TNM staging system of the International Union Against Cancer - 6^th ^Edition[20].

### Immunohistochemical detection of lymphatic vessels

4 μm formalin-fixed paraffin-embedded sections were incubated overnight at 54-56°C, deparaffinized in xylene and rehydrated through decreasing graded ethanol solutions. After endogenous peroxidase activity suppression (3% hydrogen peroxide, 10 min) and antigen retrieval (boiling in 10 mM citrate buffer, pH 6.0), immunostaining was performed with D2-40 mouse monoclonal antibody against human podoplanin (M2A antigen, Covance, California, USA) (1:100 dilution, 4°C, overnight in a humid chamber). Staining was done by using the DakoCytomation Envision Plus peroxidase mouse system. The stained protein was visualized using the DAB solution (Dako), and lightly counterstained with Mayers-haematoxilyn.

To ascertain the specificity of the antibody immunoreactivity, a negative control was carried out by exclusion of the primary antibody. In this case, immunolabeling was completely abolished.

### Evaluation of staining

Quantitative analysis of the intratumoural and peritumoural lymphatic vessel density was performed by two independent observers (DGC and MVG) in a blinded fashion. Three fields with the highest lymphatic vascular density were identified (Olympus BX-51 microscope; x100 magnification), and the vessels were counted (×400 magnification) both within the tumour area (intratumoural lymphatic density; ILD) and within an area 500 μm from the tumour border (peritumoural lymphatic density; PLD). Lymphatic vessel density (LVD), regardless of the vessel location with respect to the tumour, was also considered. Lymphatic density was defined as the number of lymphatic vessels per mm^2^.

We also evaluated invasion of the podoplanin-positive lymphatic vessels by cancer cells. Lymphatic invasion (LI) was considered to be present if at least one tumour cell cluster (a tumour embolus) was clearly visible inside a podoplanin-positive vessel. Cases were classified as LI positive or negative depending on the presence or absence of tumour emboli, respectively.

### Immunofluorescence study

Ten representative cases were selected on the basis of the images of the lymphatic vessels obtained in D2-40 IHC. For double immunostaining, deparaffined and rehydrated sections were rinsed in 0.05 M Tris-buffered saline (TBS; pH 7.5) containing 0.1% bovine serum albumin and 0.2% Triton X-100. Endogenous peroxidase activity and non-specific binding were blocked with 3% H_2_0_2 _and 50% fetal calf serum (Sigma), respectively, and the sections were incubated overnight with the primary antibodies in a humid chamber at 4°C: mouse D2-40 (M2A antigen, Covance, California, USA) and rabbit anti-ki67 (Abcam ab833). After the incubation with the primary antibodies, sections were incubated for 1 hr at room temperature with Texas red-labelled sheep anti-rabbit immunoglobulin G (IgG) and fluorescein isothiocyanate-conjugated sheep anti-mouse IgG, both diluted at 1:100 (Amersham). Finally, tissue sections were photographed with a confocal-laser scanning microscope (Bio-Rad MR-600; Servicio de Proceso de Imágenes de la Universidad de Oviedo).

### Statistical analysis

For statistical purposes, clinicopathological features were dichotomized as: pT category: 1-2, 3-4; pN category: N0, N1-3; TNM stage: I-II, III-IV. The molecular data distributed among the different clinical groups of tumours were tested for significance employing the χ^2 ^test for categorical variables and t-Student/ANOVA or Mann Whitney/Kruskal-Wallis tests for parametric and non parametric continuous variables, respectively. Survival curves (disease-specific and overall) were calculated using the Kaplan-Meier product limit estimate. Differences between survival times were analyzed by the log-rank method. Multivariate Cox proportional hazards models were used to examine the relative impact of either variables demonstrated to be statistically significant in univariate analysis or those variables likely to have an effect on the outcome. P-values < 0.05 (based on two-tailed statistical analysis) were considered statistically significant. All the statistical analyses were performed with the statistical software SPSS 12.0 (SPSS, Inc., Chicago, IL).

## Results

Results are reported following the REMARK guidelines (REporting recommendations for tumor MARKer prognostic studies)[12].

### Lymphatic density quantification

**Figure 1:**
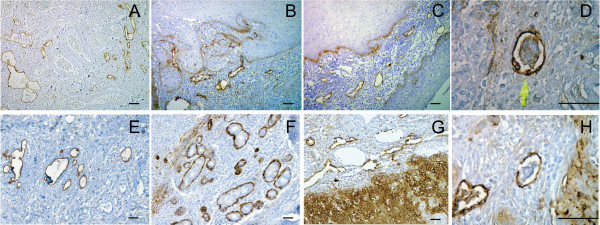
**Immunohistochemical identification of lymphatic vessels**. Immunohistochemical identification of lymphatic vessels using D2-40 antibody against podoplanin in an oropharyngeal (A) and a supraglottic tumour (E). Detection of intratumoural lymphatics in an oropharyngeal (B) and a supraglottic tumour (F). Detection of peritumoural lymphatics in a supraglottic tumour. The basal layer of the epithelium and the margin of the tumour mass gave positive signal for podoplanin (C). Detection of peritumoural lymphatics in a glottic tumour that stained positive for podoplanin (G). Example of one representative podoplanin positive tumour cell embolus inside a podoplanin positive lymphatic vessel of an oropharyngeal (D) and a supraglottic carcinoma (H). Scale bar, 250 μm.

Immunohistochemical detection of podoplanin with D2-40 antibody shows strong specific reactivity with lymphatic endothelial cells, while the endothelial cells of blood vessels show no signal (Figure 1A, E).

D2-40-positive lymphatic vessels could be identified between islands of neoplastic epithelium (Figure 1B, F) and at the tumour periphery (Figure 1C, G). Podoplanin expression was also detected in tumour cells at the tumour border (Figure 1B-C).

The percentages of the cases with positive lymphatic vessel stainings, locations of the positive standings (ILD, PLD or LVD), mean values, and score ranges in the 104 HNSCC, 54 laryngeal and 50 pharyngeal carcinoma samples are summarized in Tables 2, 3 and 4.

**Table 2:** Correlations between lymphatic vessel density and clinicopathologic features in the HNSCC series (N = 104).

ALL CASES	ILD			PLD			LVD			Lymphatic Invasion			
**Frequency**	75% (78/104)			80% (83/104)			94% (98/104)			42.72% (44/103)			
**Mean (S.E.)**	14.8 (1.33)			22.0 (1.59)			22.3 (1.19)						
**Score range**	0-78.4			0-82.2			0-79.3						

	**N**	**Mean range**	**p^#^**	**N**	**Mean (S.E)**	**p^¥^**	**N**	**Mean (S.E)**	**p^¥^**	**Absent**	**Present**	**Total**	p*

**Anatomic site**													
Pharynx	50	63.34	0.0004	50	27.41 (2.57)	0.0072	50	26.47 (1.92)	0.0029	21	29	50	0.002
Larynx	54	42.46		54	18.47 (2.03)		54	18.98 (1.56)		38	15	53	
Total	104			104			104			59	44	103	

**pT**													
T1-2	39	39.09	0.0000	39	19.19 (2.47)	0.099	39	19.20 (2.03)	0.039	29	10	39	0.006
T3-4	65	60.55		65	24.91 (2.21)		65	24.61 (1.60)		30	34	64	
Total	104			104			104			59	44	103	

**Nodal involvement**													
Free	47	42.44	0.0020	47	18.60 (2.37)	0.0230	47	19.34 (1.67)	0.0190	35	12	47	0.001
Affected	57	60.80		57	26.20 (2.27)		57	25.24 (1.82)		24	32	56	
Total	104			104			104			59	44	103	

**Tumour Stage**													
I-II	28	32.98	0.0001	28	14.67 (2.44)	0.0010	28	16.89 (2.25)	0.0060	24	4	28	0.0000
III-IV	71	56.71		71	25.83 (2.14)		71	24.76 (1.57)		31	38	69	
Total	99			99			99			55	42	97	

**Recurrence**													
No	66	50.98	0.8167	66	20.29 (2.17)	0.0729	66	21.15 (1.54)	0.1649	38	27	65	0.8050
Yes	34	49.57		34	26.86 (2.78)		34	25.04 (2.47)		19	15	34	
Total	100			100			100			57	42	99	

ILD, Intratumoural Lymphatic Density; PLD, Peritumoural Lymphatic Density; LVD, Lymphatic Vessel Density

S.E., Standard Error

p^# ^(U Mann-Whitney); p^¥ ^(t Student); p* (chi square)

Frequencies (cases with lymph vessels), mean values and score ranges of ILD, PLD and LVD in the whole series (N=104). Comparison of mean ILD, PLD and LVD values among different categories of clinico-pathological features. Distribution of tumours according to lymphatic invasion (LI) and clinicopathological features.

**Table 3:** Correlations between lymphatic vessel density and clinicopathologic features in the laryngeal carcinoma series (N = 54).

LARYNX	ILD			PLD			LVD)			Lymphatic Invasion			
**Frequency**	57.41% (31/54)			55.56% (30/54)			87.04% (47/54)			28.30% (15/53)			
**Mean (S.E.)**	10.59 (1.65)			18.47 (2.03)			18.98 (1.56)						
**Score range**	0-41.56			0-60.45			0-44.71						

	**N**	**Mean range**	**p#**	**N**	**Mean (S.E)**	**p¥**	**N**	**Mean (S.E)**	**p¥**	**Absent**	**Present**	**Total**	p*

**Anatomic site**													
Glottis	30	23.45	0.028	30	15.22 (2.43)	0.073	30	16.14 (2.06)	0.042	23	6	29	0.176
Supraglottis	24	32.56		24	22.54 (3.30)		24	22.52 (2.24)		15	9	24	
Total	54			54			54			38	15	53	

**pT**													
T1-2	28	22.05	0.006	28	15.95 (2.85)	0.2	28	15.98 (2.38)	0.044	25	3	28	0.003
T3-4	26	33.37		26	21.19 (2.86)		26	22.20 (1.84)		13	12	25	
Total	54			54			54			38	15	53	

**Nodal involvement**													
Free	38	25.38	0.112	38	16.63 (2.23)	0.165	38	17.72 (1.81)	0.219	30	8	38	0.062
Affected	16	32.53		16	22.85 (4.27)		16	21.96 (3.03)		8	7	15	
Total	54			54			54			38	15	53	

**Tumour Stage**													
I-II	24	20.92	0.004	24	13.95 (2.52)	0.045	24	15.05 (2.35)	0.023	20	4	24	0.073
III-IV	30	32.77		30	22.09 (2.92)		30	22.12 (1.94)		17	11	28	
Total	54			54			54			37	15	52	

**Recurrence**													
No	43	28.35	0.169	43	17.02 (2.28)	0.218	43	18.60 (1.80)	0.685	29	13	42	0.492
Yes	10	21.20		10	23.55 (4.7)		10	20.28 (3.57)		8	2	10	
Total	53			53			53			37	15	52	

ILD, Intratumoural Lymphatic Density; PLD, Peritumoural Lymphatic Density; LVD, Lymphatic Vessel Density

S.E., Standard Error

p^# ^(U Mann-Whitney); p^¥ ^(t Student); p* (chi square)

Frequencies (cases with lymph vessels), mean values and score ranges of ILD, PLD and LVD in laryngeal carcinoma population (N=54). Comparison of mean ILD, PLD and LVD values among different categories of clinico-pathological features. Distribution of tumours according to lymphatic invasion (LI) and clinicopathological features.

**Table 4:** Correlations between lymphatic vessel density and clinicopathologic features in the laryngeal carcinoma series (N = 50).

PHARYNX	ILD			PLD			LVD			Lymphatic Invasion			
**Frequency**	90% (45/50)			90% (45/50)			96% (48/50)			58% (29/50)			
**Mean (S.E.)**	19.14 (1.97)			27.41 (2.57)			26.47 (1.92)						
**Score range**	0-78.39			0-82.17			8.5 -79.34						

	**N**	**Mean (S.E)**	**p¥**	**N**	**Mean (S.E)**	**p¥**	**N**	**Mean range**	**p#**	**Absent**	**Present**	**Total**	p*

**Anatomic site**													
Hypopharynx	18	19.92 (3.48)	0.8600	18	30.87 (3.91)	0.3470	18	29.67	0.0810	12	6	18	0.0230
Oropharynx	31	19.18 (2.45)		31	25.70 (3.46)		31	22.29		9	22	31	
Total	49			49			49			21	28	49	

**pT**													
T1-2	11	14.71 (3.53)	0.236	11	27.45 (3.12)	0.993	11	29.18	0.343	4	7	11	0.668
T3-4	39	20.39 (2.30)		39	27.39 (3.11)		39	24.46		17	22	39	
Total	50			50			50			21	29	50	

**Nodal involvement**													
Free	9	16.30 (3.90)	0.3330	9	26.94 (7.77)	0.3450	9	27.50	0.6550	5	4	9	0.3630
Affected	41	19.77 (2.25)		41	27.51 (2.69)		41	25.06		16	25	41	
Total	50			50			50			21	29	50	

**Tumour Stage**													
I-II	4	12.12 (7.01)	0.3720	4	19.05 (8.74)	0.3650	4	28.13	0.4280	4	0	4	0.0100
III-IV	41	19.60 (2.29)		41	28.57 (2.99)		41	22.50		14	27	41	
Total	45			45			45			18	27	45	

**Recurrence**													
No	23	19.19 (2.37)	0.9140	23	26.41 (4.34)	0.7410	23	24.39	0.8480	9	14	23	0.6420
Yes	24	18.73 (3.45)		24	28.24 (3.43)		24	23.63		11	13	24	
Total	47			47			47			20	27	47	

ILD, Intratumoural Lymphatic Density; PLD, Peritumoural Lymphatic Density; LVD, Lymphatic Vessel Density

S.E., Standard Error

p^# ^(U Mann-Whitney); p^¥ ^(t Student); p* (chi square)

Frequencies (cases with lymph vessels), mean values and score ranges of ILD, PLD and LVD in pharyngeal population (N=50). Comparison of mean ILD, PLD and LVD values among different categories of clinico-pathological features. Distribution of tumours according to lymphatic invasion (LI) and clinicopathological features.

### Proliferating lymph vessels

**Figure 2:**
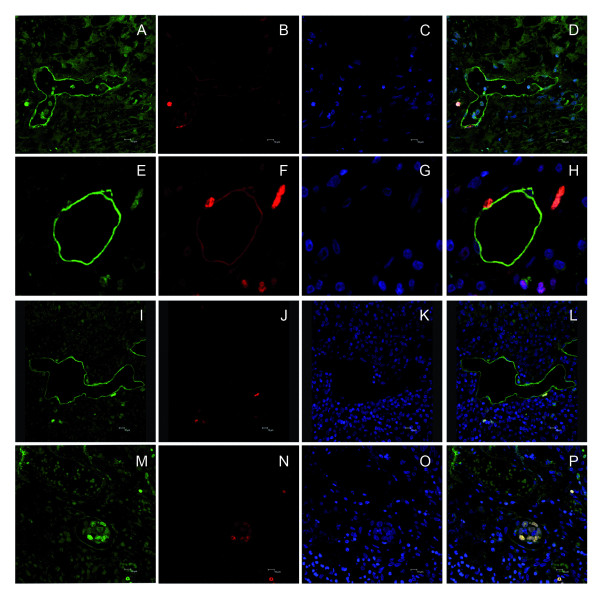
**Proliferation state of lymphatic vessels**. Examples of podoplanin positive (green) proliferative (ki-67 positive, red) lymphatic vessels in an oropharyngeal (A-D and I-L) and a supraglottic carcinoma (E-H). D2-40 positive (green) proliferating tumour mass inside a lymphatic vessel of a supraglottic carcinoma (M-P) (lymphatic invasion positive case, ki-67 positive embolus, red). First and second columns D2-40 -green- and ki-67 -red- immunstainings, respectively; third and last columns, DAPI (blue) and merged images. Scale bar, 10 μm.

Double immunofluorescence assay was performed with D2-40 and the proliferation marker ki-67 in ten representative cases. Vessels were deemed positive for proliferation when they harboured at least one ki-67 positive cell (Figure 2A-L) and were observed inside the tumour mass or in its periphery.

### Lymphatic invasion (LI)

Podoplanin-positive carcinoma cells could be visualized inside lymph vessels (irrespective of vessel location) in 43% of the cases (44/103, LI positive) (Figure 1D, H). Figure 2M-P shows the proliferation state (ki-67) of a D2-40 positive tumour mass inside a lymphatic vessel (LI positive case).

### Correlations with clinicopathologic features

Considering the whole series for analysis, higher mean ILD, PLD and LVD values were significantly associated with greater tumour size, lymph node metastasis, advanced tumour stage and pharyngeal tumour site (Table 2). Tables 3 and 4 summarize ILD, PLD and LVD mean values and LI distribution among clinicopathological features in laryngeal and pharyngeal carcinomas, respectively. Of note, while no association was found in the pharyngeal group, LVD correlated with greater tumour size and more advanced tumour stage within the laryngeal population (p < 0.05, each).

The normal glottis is a particularly interesting site, since it is a lymph vessel free region. In our series, 13 out of the 29 glottic cases (45%) showed lymphatics within the tumour mass, 3 of which were T1 tumours.

Importantly, the presence of tumour emboli (lymphatic invasion) correlated with anatomic site (28% in laryngeal tumours *vs*. 58% in pharyngeal tumours; p = 0.002) and were more frequently observed in tumours of patients with affected lymph nodes (p = 0.001), in greater size tumours (p = 0.006) and in more advanced stage tumours (p = 0.000).

### Survival analysis

The five-year disease specific and overall survival rates for the whole population were 52% and 28%, respectively. As expected, tumours arising in the larynx, those with T1-2 category, stage I-II and patients without lymph node involvement, had a better prognosis (p < 0.05).

**Figure 3:**
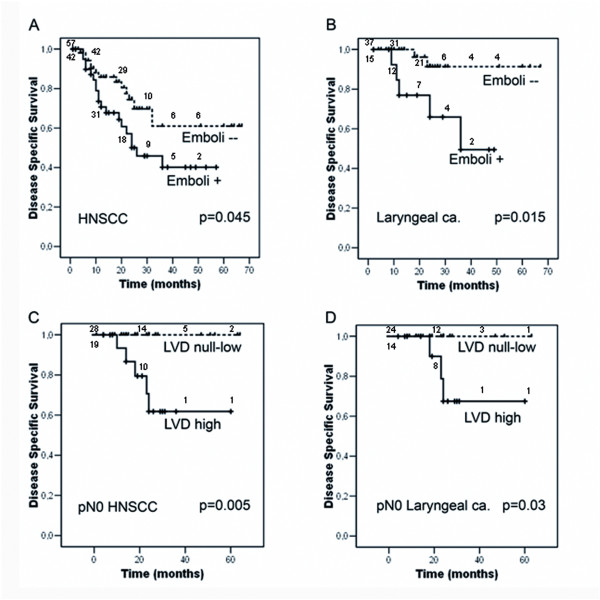
**Lymphatic Vessel Density and survival**. Disease specific survival curves for pharyngolaryngeal squamous carcinoma patients (A) or laryngeal carcinoma patients (B) stratified by the presence of tumour emboli inside lymphatics. pN0 HNSCC patients (C) or pN0 laryngeal carcinoma (D) stratified according to LVD (null-low *vs*. high). The number of patients remaining after every ten/twenty months period is indicated along each curve. Log-rank test significance is indicated.

For the survival analysis, lymphatic vessel variables (ILD, PLD, LVD) were categorized into high vessel density cases *vs*. the rest, attending to the median value of the lymph vessel density in the positive population (Table 5). In this way, a high LVD (irrespective of vessel location) had a negative impact on disease specific survival (p = 0.045) and on overall survival (p = 0.0564). In addition, disease specific (Figure 3A) and overall survival were worse for patients with tumour emboli within the vessels (LI positive) (p = 0.045 and p = 0.0312, respectively). Importantly, these results were observed for the laryngeal tumours (high LVD (p = 0.027), presence of emboli; p = 0.015) (Figure 3B) but not in the pharyngeal ones.

**Table 5:** Impact of the lymphatic vessel density on survival.

	ALL CASES (N = 104)		Larynx (N = 54)		Pharynx (N = 50)	
	
	Median	p	Median	p	Median	p
ILD	13.22	0.15	7.56	0.3262	18.42	0.6831
PLD	22.67	0.3898	20.78	0.6321	26.92	0.7139
LVD	22.04	**0.045**	20.78	**0.027**	22.98	0.334
LI		**0.045**		**0.015**		0.5933

Kaplan Meier disease specific survival analysis p-values for ILD, PLD, LVD - dichotomized according to their median values - in the whole population, laryngeal and pharyngeal tumours

A very interesting observation could be made for the 47 HNSCC patients with pN0 tumours (38 laryngeal and 9 pharyngeal): 19 cases showing a high LVD (14 laryngeal and 5 pharyngeal) had a poorer prognosis than the rest (p = 0.005) (Figure 3C). Among the group of 38 pN0 laryngeal tumours a high LVD was the only marker of poor prognosis in univariate analysis (p = 0.03) (Figure 3D).

None of these variables reached statistical significance when multivariate Cox analysis was performed.

## Discussion

In the present study we investigated the existence of tumour lymphangiogenesis in a cohort of 104 laryngeal/pharyngeal carcinoma patients and evaluated whether ILD, PLD or LVD correlated with the presence of lymph node metastasis; we also addressed the prognostic significance of the above parameters on the disease specific and overall survival of these patients.

As previously described, podoplanin was detected in tumour cells [13] and lymphatic vessels were observed both within the tumour mass (ILD) and in the peritumoural area (PLD)[5,14-17]. Higher ILD correlated with a more aggressive tumour phenotype and high LVD was predictor of poor survival (p = 0.045). However, when laryngeal and pharyngeal tumours were considered separately, these associations were observed only in laryngeal carcinomas (LVD and survival, p = 0.027), suggesting a different contribution of lymphangiogenesis at different anatomic sites. While heterogeneous results can be found in the literature regarding this question,[5,14-17] our data leave evidence of a more relevant role of lymphangiogenesis in the laryngeal carcinoma subgroup. Anatomic characteristics could account for these differences. The pharynx is a lymph vessel rich area so that virtually all tumours arising there might benefit from this circumstance. In our series 96% pharyngeal tumours were LVD positive and 90% ILD positive. Thus, no differences can be expected to be observed regarding lymph vessel density impact on outcome. In contrast, the larynx (specially the glottis) is a lymph vessel poor region and those tumours able to induce lymphangiogenesis (57% ILD positive cases in our series) might show a selective advantage regarding spread and thus a poorer outcome than those that remain vessel free.

In reference to the existence of dividing cells among lymphatic endothelial cells, we found evidence of at least one proliferative endothelial lymphatic cell in some tumour sections, although their proportion was low. These results are in line with previous reports that point to the existence of an active lymphangiogenesis process in HNSCC[5,15].

The correlation between ILD and nodal involvement supports the role of intratumoural lymph vessels as a possible route for the spread of tumours to local lymph nodes. Interestingly, tumour emboli (LI, lymphatic invasion) were observed within lymphatics in 43% of tumour samples, a higher frequency than previously reported[5,15,17,18]. Importantly, LI correlated with tumour size, nodal involvement, tumour stage and a reduced survival rate.

Lymphatic cell proliferation and tumour emboli formation may be rare events, difficult to detect by examining small sections of archival tissue at a single time point. Despite this, the detection of proliferating lymphatics in carcinoma tissue and of tumour emboli within lymphatics constitutes considerable evidence for the presence of active lymphangiogenesis in this type of malignancy. Additionally, we present evidence of a true lymphangiogenic process in the larynx, since lymphatics were detected in pT1 tumours arising in the glottis, a physiologically lymph vessel free site. This observation would indicate the formation of new vessels in these cases. On the other hand, the presence of lymphatics in the pT2-4 glottic tumours would not necessarily point to this phenomenon, since vessels might also result from their being entrapped as growing tumours reached the vessel rich areas, such as the paraglottic space, supraglottis or subglottis.

Controversial results can be found in the literature regarding the specific contribution of intra and peritumoural lymphatic vessels to tumour spread. Functional studies in animal models have shown that intratumoural lymphatic vessels are compressed and nonfunctional. However, some studies on human tumour samples reveal that intratumoural lymphatics are involved in nodal metastasis and that ILD is a significant indicator of poor prognosis[5,14,17]. Other authors describe an association of high PLD with shorter disease-free survival,[16,19] whereas a correlation between higher PLD and a more favourable outcome has also been reported[17]. These heterogeneous results could arise from the different methods used to quantify LVD and could reflect subjective factors and the intrinsic difficulties found in intra *vs*. peritumoural vessel identification when studying single tissue sections taken at a single time point, which might yield a misleading vessel classification, limiting the potential value of these variables. Although both ILD and PLD contribute to tumour spread to lymph nodes, from our experience, LVD counting (regardless of their location with respect to the tumour) is more reproducible and might be a more reliable marker of poor prognosis, especially within the laryngeal subgroup.

Remarkably, although nodal involvement had a negative prognostic value, this prediction could be refined among the pN0 laryngeal tumour population, according to their LVD. Thus, significant differences on survival times were found for cases showing a high LVD (poorer outcome). This observation was also made in the whole series of carcinomas but not in the pharyngeal subgroup, and might be a useful tool in the pN0 laryngeal cancer patients' clinical management.

## Conclusions

In summary, our results suggest a differential implication of lymphangiogenesis in tumours arising from different anatomical head and neck sites. We propose that LVD assessment and tumour emboli detection using the D2-40 antibody should be considered as potential prognostic tools for laryngeal tumours. This would merit further large-scale validation analyses for the eventual future incorporation of these potential markers into clinical practice.

## Competing interests

The authors declare that they have no competing interests.

## Authors' contributions

DGC carried out the IHC studies and vessel counting and helped to draft the manuscript. JPR participated in its design and contributed to clinical data collection and results interpretation. AA contributed to the pathological assessment of tissue sections. CSN participated in its design and coordination. MVG conceived the study, coordinated and supervised its progression, carried out vessel counting, performed the statistical analysis and wrote the manuscript. All authors read and approved the final manuscript.

## Pre-publication history

The pre-publication history for this paper can be accessed here:

http://www.biomedcentral.com/1471-2407/10/416/prepub
